# Scheduling non-critical activities using multicriteria approach

**DOI:** 10.1007/s10100-018-0542-y

**Published:** 2018-04-27

**Authors:** Krzysztof S. Targiel, Maciej Nowak, Tadeusz Trzaskalik

**Affiliations:** Department of Operations Research, University of Economics in Katowice, ul. 1 Maja 50, 40-287 Katowice, Poland

**Keywords:** Project scheduling, Trade-offs, Interactive approach, CRR binomial method

## Abstract

In many projects the problem of selecting the start time of a non-critical activity arises. Usually it is possible to use the “as soon as possible” or “as late as possible” rules. In some situations, however, the result of such a decision depends on external factors such as exchange rate. This leads to an approach in which the problem of scheduling non-critical activities is solved using an expanded Cox–Ross–Rubinstein (CRR) binomial tree method. In the paper a bi-criteria problem of determining the start time of a non-critical activity is considered. We assume that the early start and the late start of the activity have been identified using Critical Path Method, but the project manager is free to select the time when the activity will actually be started. This decision cannot, however, be changed later, as it is associated with the allocation of key resources. Two main criteria are considered: cost and risk. While cost depends on exchange rate, risk increases with the delay of the start of the activity. The problem can be described as a dynamic process. We propose a new interactive technique for solving such a bi-criteria decision making problem under risk. The procedure uses trade-offs to identify a candidate solution. The CRR binomial method is applied to evaluate the cost of the activity.

## Introduction

The Critical Path Method (CPM) was developed in the late 1950s by Kelley and Walker (1959). Despite the passage of over 50 years, the CPM method is still being mentioned as the main tool for creating schedules. The last edition of PMBoK (PMI 2017) refers to it in the discussion of the process Develop schedule, described in Project Schedule Management Knowledge Area. CPM divides activities in each project into two categories: critical and non-critical. Critical activities are those for which the start and finish times are strictly defined. They are critical in the sense that their delay results in the delay of the whole project. The start time for non-critical activities can, to a certain extent, be freely selected. Classical project scheduling methods (CPM, PERT) are still among the most frequently used operations research tools (Cvetkoska 2016).

In the literature, little attention is paid to non-critical activities. Castro et al. (2008) consider this problem while analyzing the slack allocation in PERT. They propose an allocation rule that assigns extra time to the activities proportionally to their durations in such a way that no path duration exceeds the completion time of the whole project. Similar problem, extended to a stochastic framework, is also considered in Castro et al. (2014). Although defining a critical path is extremely important for project management, in the literature little attention is paid to the identification of such a path in the PERT model. This problem is considered by Monhor (2011), who proposes a new probabilistic approach for comparing path durations and defines the concept of probabilistically critical path as a stochastic counterpart of the deterministic critical path.

In practical applications, deterministic estimation is usually used for activity duration. However, even in this situation, the project manager faces the problem of determining when a non-critical activity should start. Two approaches are usually proposed to solve this problem:

- As Soon As Possible (ASAP)
- As Late As Possible (ALAP)

**Fig. 1:**
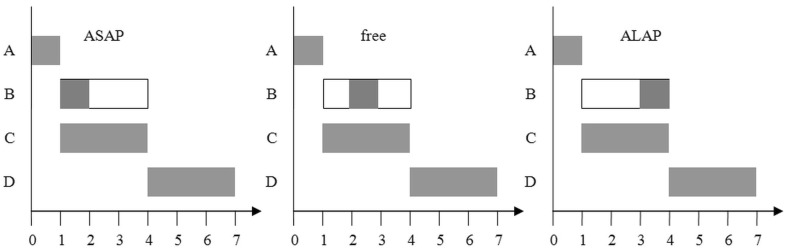
Approaches to the choice of when to start a non-critical activity (B)

Figure 1 describes these approaches, assuming that B is a non-critical activity, while the others are critical.

The ASAP approach is more appropriate when it is very important to meet the project deadline. This method minimizes the risk of exceeding this date. On the other hand, ALAP, the more risky approach, can be selected to meet resource constraints. There is also a third option: to start a non-critical activity between these extremes. The aim of this paper is to propose a new method for solving this problem.

In some cases the result of an activity depends on its finish time. This is the case, for example, of construction projects where the activity cost depends on the cost of building materials which varies seasonally. In this situation, choosing the right moment to start a non-critical activity is of major importance. This is an interesting research problem which raises the question of whether it is possible to determine the optimal start time using the history of changes in the factors that determine the result of the activity. Papageorgiou (2013) mentions this problem, stating that using real options may give some flexibility in managing non-critical activities in R&D projects.

Financial literature proposes various solutions for a similar problem, called the timing problem, based on the valuation of financial options. A well-known solution is the Cox–Ross–Rubinstein (CRR) method (Cox et al. 1979), based on the binomial tree. Uncertainty in project financial evaluation was also discussed by Gaspars-Wieloch (2017), who applied the Net Present Value (NPV) model, which is very similar to the binomial tree approach.

Scheduling problems are often considered in the multicriteria framework. Most techniques proposed in the literature are dedicated to the Resource Constrained Project Scheduling Problem (RCPSP). As such a problem is NP-hard, metaheuristic approaches are usually used (Monghasemi et al. 2015; Tofighian and Naderi 2015; Ning et al. 2017; Palacio and Larrea 2017). However, in this study we assume that resource allocation is not a problem for the decision maker. Our problem is to determine the start time of the activity, taking into account its cost and the probability of delay. This corresponds, for example, to the situation of the general contractor who hires a subcontractor to perform an activity.

In this paper the selection of the start time of a non-critical activity is formulated as a dynamic bi-criteria decision making problem under risk. The first criterion is the expected cost of the activity, the second, the probability of delay. This problem was described previously by Targiel (2015), who used the TOPSIS method. The main contribution of this paper is the solution of the problem by means of the proposed interactive procedure.

Solving a multicriteria problem requires information about the decision-makers preferences. Two main approaches can be used to aquire it (Roy 1971). The first assumes that the decision-maker gives his/her preference information on an a priori basis. The procedure is divided into two distinct phases: (1) preference information acquisition, (2) computations. This approach is often criticized. First, the decision-maker has to consider all kinds of choices and trade-offs which might be relevant, and as this information is acquired before knowing whether the alternatives are influenced by these preferences, it may be redundant. Moreover, the decision-maker may find the choices he/she faces as purely hypothetical, which results in his/her reduced concentration, thereby reducing the quality of the information obtained.

The interactive approach is an alternative to methods based on an a priori basis. In this approach preference information is acquired step by step. In each iteration the dialogue and computational phases are repeated. The decision-maker is more closely involved in the process of solving the decision problem, and as a result, improves his/her knowledge about the structure of the problem.

Two main paradigms are used for identifying preference information: direct and indirect (Kaliszewski et al. 2012). The former assumes that the decision-maker expresses his/her preferences in relation to the values of attributes. This approach is used e.g. by Benayoun et al. (1971). Indirect collection of information on preferences means that the decision-maker has to determine the trade-offs among attributes in each iteration, given the current candidate solution. The method proposed by Geoffrion et al. (1972) is an example of this approach. Techniques combining both approaches have been also proposed, for instance in Kaliszewski and Michalowski (1999). As was shown in Nowak (2010), trade-offs can also be used to solve a discrete stochastic multicriteria decision making problem.

Interactive techniques can also be applied to solve dynamic decision making problems (Nowak and Trzaskalik 2013; Nowak 2016; Nowak et al. 2017). However, most techniques dedicated to dynamic problems use the direct paradigm to identify preference information. In this paper we propose a procedure using trade-offs to identify a candidate solution. The CRR binomial method is applied to evaluate the cost of the activity.

## Problem formulation

Let us assume that the cost of the activity is expressed in foreign currency (e.g. EUR). The cost in domestic currency (e.g. PLN) depends on the exchange rate, which fluctuates constantly. Since we assume that the activity is non-critical, the problem of selecting its start time arises. If the probability that the exchange rate will fall is higher than the probability of its increase, it is quite clear that the activity should be started as late as possible. On the other hand, the later the activity is started, the higher the risk that it will not be completed on time. Our assumptions are as follows:

1. The cost of the activity is expressed in foreign currency, and does not depend on the actual completion time.
2. The minimal completion time ($t_{min}$) and the latest finish (*LF*) for the activity have been estimated.
3. For organizational reasons the activity can be started only at the beginning of the successive periods: $n = 1, 2, \ldots , LF - t_{min}$.
4. The actual cost of the activity in domestic currency depends on the exchange rate at the end of the period in which the activity is started.
5. For each *n*, expert estimations of the probability that the activity is finished on time, assuming that it is started at the beginning of period *n*, are available.
6. The problem consists in deciding when to start the activity, taking into account two criteria: $f_1$ the cost of the activity, and $f_2$ the probability that the activity is delayed.

**Fig. 2:**
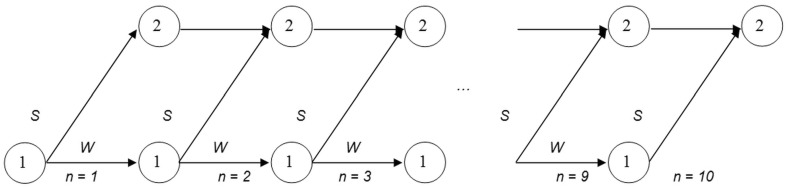
Dynamic process

Our problem can be described as a dynamic process consisting of *N* periods, where $N=LF - t_{min}$ (Fig. 2). At the beginning of the first period the process is in state (1), while at the beginning of the other periods it can be in state (1) or (2), where state (1) means that the activity has not been started yet, while state (2) means that the activity has already been started. Thus, two decisions can be made in state (1): *S*—start the activity, and *W*—wait. The only decision in state (2) is to continue the process.

In our problem *N* alternatives, representing the period in which the activity is started, are considered.

## Bi-criteria procedure for scheduling non-critical activities

### Modelling future with a binomial tree

We assume that the future value of our parameter of interest (*X*) can be modelled using stochastic differential equations. For this purpose we choose Geometric Brownian Motion (GBM) with the equation:

1 $$\begin{aligned} dX(t) = \mu X(t) dt + \sigma X(t) dW(t), \end{aligned}$$

where *W*(*t*) is the Wiener process, *X*(*t*) is the value of parameter *X*, $\mu$ is the drift parameter, $\sigma$ is the volatility parameter.

**Fig. 3:**
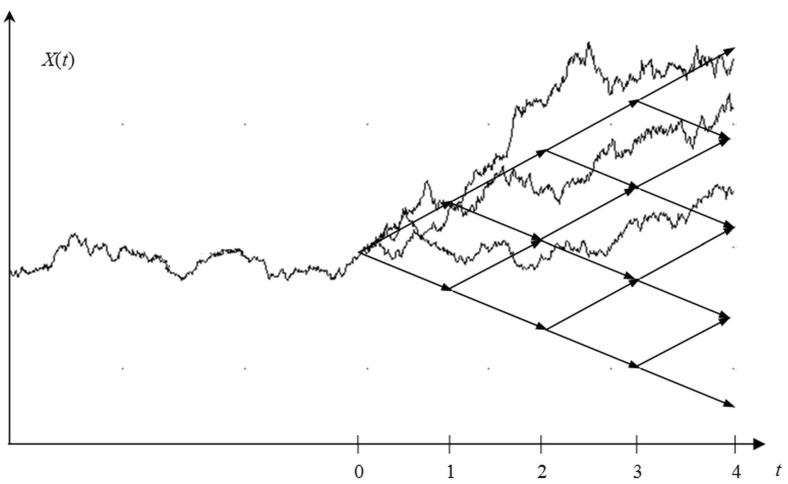
A binomial tree covering the stochastic process

An implementation of this process is shown in Fig. 3 up to point $t = 0$.

This continuous process can be approximated by a discrete structure, namely the binomial tree. This approach was discussed in Targiel (2013). The nodes of this graph can be calculated from the formula:

2 $$\begin{aligned} x_{i,n} = x_{0,0} e^{(n-2i)\hat{\sigma } \sqrt{\varDelta t_p}} \end{aligned}$$

where $x_{i,n}$ is the value of parameter *x* after *n* periods and *i* declines, $\varDelta t_p$ is the part of the year which represents one period in tree, $\hat{\sigma }$ is the estimated volatility parameter for GBM.

We can estimate such parameters on the basis of historical data. The estimated volatility $\hat{\sigma }$ of the process is calculated on the basis of historical data from their variability:

3 $$\begin{aligned} \hat{\sigma } = \frac{\sigma _d }{\sqrt{\varDelta t_d}} \end{aligned}$$

where $\varDelta t_d$ is the part of the year which represents one period in data and $\sigma _d$ is the standard deviation in historical data.

Knowing that, we can calculate the typical growth factor (*u*) (with the fall factor 1 / *u*):

4 $$\begin{aligned} u = e^{\hat{\sigma } \sqrt{\varDelta t_p}} \end{aligned}$$

The probability of increase can be calculated using the following formula:

5 $$\begin{aligned} q = \frac{1}{2} + \frac{\mu \sqrt{\varDelta t_m}}{2 \hat{\sigma }} \end{aligned}$$

We can also calculate the probability of reaching each node (*i*, *n*) after *n* periods (Guthrie 2009):

6 $$\begin{aligned} P\left\{ x \, at \, (i, n) \right\} = \frac{n!}{i!(n-i)!}q^{n-i}(1-q)^i \end{aligned}$$

This leads us directly to the expected value of parameter *X* at each stage *n*:

7 $$\begin{aligned} \mathbf {E}[X(n)] = \sum _{i=0}^{N}{\frac{n!}{i!(n-i)!}q^{n-i}(1-q)^i}x_{i,n} \end{aligned}$$

With formula (7) we can calculate the expected cost of the activity which will start at each moment n, which gives us the first criterion:

8 $$\begin{aligned} f_1(a^{(n)}) = K\mathbf {E}[X(n)] \end{aligned}$$

where *K* denotes the fixed cost in EUR, and parameter *X* is the exchange rate EUR/PLN.

### Modelling risk

The second criterion is risk, measured as the probability of delay. A non-critical activity must be finished before a specific point in time. Its delay causes a delay of the entire project. This probability can be derived from the expected duration estimated using the PERT method (Program Evaluation and Review Technique) (Stauber et al. 1959), but it is more useful to approach this calculation with expert knowledge and intuition. For each alternative we ask an expert to define the probability of delay. This approach allows to take into account the risk associated with weather which is very important for construction projects. In the example presented below it is assumed that a non-critical activity can be started between January and October (Table 1). When the activity is started in January (alternative $a^{(1)}$), there is 1% chance of its delay past the end of the year, but when it starts in October (alternative $a^{(10)}$), there is 20% chance that not only the given activity but also the entire project will be delayed.

### Interactive approach

The procedure proposed here can be used to solve a discrete bi-criteria problem with up to a moderate number of alternatives (not more than 100). By A we denote the set of alternatives. In our problem, each alternative represents the period in which the non-critical activity is started. We also assume that both criteria are minimized.

In the initial phase of our procedure, the set of non-dominated alternatives $\mathbf {A}^{*}$ is identified. An alternative *a* is non-dominated if there is no $a'$ such that (Roy 1985):

$f_1(a') \le f_1(a)$ and $f_2(a') < f_2(a)$ or $f_1(a') < f_1(a)$ and $f_2(a') \le f_2(a)$

In each iteration, a candidate alternative $a^*$ is proposed to the decision-maker, who is asked whether the values of criteria are satisfactory. If the answer is ‘yes’, the procedure ends. Otherwise, a new candidate is identified. We propose here to use trade-offs for identifying the new candidate. This is a ratio representing the value by which one criterion is improved by the unit deterioration of the reference criterion, when alternative *a* is replaced by $a^*$.

Let us assume that alternative $a^*$ is presented to the decision maker and he/she states that the value of $f_1$ is too high. In this case we look for alternative *a* which maximizes the value of the following ratio:

9 $$\begin{aligned} T_{1,2}(a, a^*) = \frac{f_1(a^*) - f_1(a)}{f_2(a)-f_2(a^*)} \end{aligned}$$

In this case, the trade-off measures the decrease in cost by one unit of increase in the probability of delay. On the other hand, if the decision-maker finds the probability of delay unsatisfactory, a new candidate maximizing the following trade-off is identified:

10 $$\begin{aligned} T_{2,1}(a, a^*) = \frac{f_2(a^*) - f_2(a)}{f_1(a)-f_1(a^*)} \end{aligned}$$

The data presented to the decision maker in each iteration include: the evaluations of the proposed alternative and the potency matrix. The latter consists of two rows grouping optimistic and pessimistic values of criteria attainable within the whole set of non-dominated alternatives. As we assume that both criteria are minimized, the following formulas can be used for calculating optimistic ($\overline{f}_1$ and $\overline{f}_2$) and pessimistic values ($\underline{f}_1$ and $\underline{f}_2$):

$$\begin{aligned} \overline{f}_1:= & {} \min _{{a \in \mathbf {A}^{*}}} \{ f_1(a) \} \qquad \overline{f}_2 := \min _{{a \in \mathbf {A}^{*}}} \{ f_2(a) \}\\ \underline{f}_1:= & {} \max _{{a \in \mathbf {A}^{*}}} \{ f_1(a) \} \qquad \underline{f}_2 := \max _{{a \in \mathbf {A}^{*}}} \{ f_2(a) \} \end{aligned}$$

The procedure operates as follows:

*Initial phase*

1. For each $a \in \mathbf {A}$ calculate the expected cost using formula (8).
2. Identify the set of non-dominated alternatives $\mathbf {A}^{*}$.
3. Identify the first candidate $a^*$:
  $$\begin{aligned} a^* := arg \min _{{a \in \mathbf {A}^{*}}} \{ f_1(a) \} \end{aligned}$$
4. Set $$l:=1$$.

*Iteration* *l*:

1. Present the results to the decision maker
  The evaluations of the candidate alternative $a^*$: $f_1(a^*)$, $f_2(a^*)$.
  The potency matrix.
2. Ask the decision maker whether he/she is satisfied with the proposal. If the answer is *yes*—go to (11).
3. Ask the decision maker whether he/she is satisfied with the value of $f_1$. If the answer is *yes*—go to (7).
4. Identify the set $\hat{\mathbf {A}}$:
  $$\begin{aligned} \hat{\mathbf {A}} := \{ a: a \in \mathbf {A}^{*}, f_1(a) < f_1(a^*) \} \end{aligned}$$
5. If $\hat{\mathbf {A}}= \varnothing$, inform the decision-maker that improving the value of $f_1$ is impossible and ask the decision maker whether he/she wants to continue the procedure. If the answer is no, go to (11), otherwise go to (2). If $\hat{\mathbf {A}} \ne \varnothing$, go to (6).
6. For each $a \in \hat{\mathbf {A}}$ calculate the trade-off using formula (9). Identify the alternative maximizing the value of the trade-off and set it to be the new candidate $a^*$. Go to (10).
7. Identify the set $\hat{\mathbf {A}}$:
  $$\begin{aligned} \hat{\mathbf {A}} := \{ a: a \in \mathbf {A}^{*}, f_2(a) < f_2(a^*) \} \end{aligned}$$
8. If $\hat{\mathbf {A}}= \varnothing$, inform the decision-maker that improving the value of $f_2$ is impossible and ask the decision maker whether he/she wants to continue the procedure. If the answer is *no*, go to (11), otherwise go to (2). If $\hat{\mathbf {A}} \ne \varnothing$, go to (9).
9. For each $a \in \hat{\mathbf {A}}$ calculate the trade-off using formula (10). Identify the alternative maximizing the value of the trade-off and set it to be the new candidate $a^*$.
10. Set $\mathbf {A}^* := \hat{\mathbf {A}}$, $l:=l+1$, and go to (1).
11. End of the procedure.

## A numerical example

We consider a non-critical activity that should be completed not later than December 31. The cost of the activity is 50 million euro and does not depend on the completion time. The initial PLN/EUR rate is 4.1472. The probability *q* of increase is 0.4. The minimal completion time is 3 months. Obviously, the sooner the activity is started, the lower the risk that the activity will be delayed. Table 1 presents the probabilities that the activity will not be completed on time.

In the initial phase, a binomial tree is used to generate the probability distributions of the PLN/EUR rate. Next, these distributions are used to identify the distributions of the activitys cost. Table 1 presents the expected costs for various starting times.

**Table 1:** The set of alternatives

Alternative	Starting month	Expected cost (PLN $10^3$)	Probability of delay
$a^{(1)}$	January	206,635	0.01
$a^{(2)}$	February	205,913	0.02
$a^{(3)}$	March	205,194	0.04
$a^{(4)}$	April	204,476	0.10
$a^{(5)}$	May	203,762	0.11
$a^{(6)}$	June	203,050	0.15
$a^{(7)}$	July	202,340	0.16
$a^{(8)}$	August	201,633	0.18
$a^{(9)}$	September	200,928	0.19
$a^{(10)}$	October	200,226	0.20

It is quite clear that all alternatives are non-dominated, since the later the activity starts, the lower the expected cost and the higher the risk of delay. Alternative $a^{(10)}$ is the one for which the expected cost is minimal and is therefore an initial proposal for the decision maker. The identification of the final solution proceeds according to the following scenario:

*Iteration 1:*

1. The potency matrix (Table 2) and the candidate solution are presented to the decision-maker: $f_1(a^{(10)}) = 200{,}226$; $f_2(a^{(10)}) = 0.20$.
2. The decision-maker is not satisfied with the proposal.
3. The decision-maker is satisfied with the expected cost. The procedure advances to step (7).
4. The set $\hat{\mathbf {A}}$ is identified:
  $$\begin{aligned} \hat{\mathbf {A}} := \left\{ a^{(1)}, a^{(2)}, a^{(3)}, a^{(4)}, a^{(5)}, a^{(6)}, a^{(7)}, a^{(8)}, a^{(9)} \right\} \end{aligned}$$
5. As the set $\hat{\mathbf {A}}$ is not empty, the procedure advances to the next step.
6. Trade-offs are calculated for $a \in \hat{\mathbf {A}}$ (Table 3). Alternative $a^{(3)}$ is identified as the new candidate solution.
7. $\mathbf {A}^* := \hat{\mathbf {A}}$, $l:=2$, and the procedure advances to the next iteration.

**Table 2:** Potency matrix in iteration 1

	Expected cost	Probability of delay
Optimistic value	200,226	0.01
Pessimistic value	206,635	0.20

**Table 3:** Trade-offs in iteration 1

Alternative	$a^{(1)}$	$a^{(2)}$	$a^{(3)}$	$a^{(4)}$	$a^{(5)}$	$a^{(6)}$	$a^{(7)}$	$a^{(8)}$	$a^{(9)}$
Trade-off	0.0296	0.0317	0.0322	0.0235	0.0255	0.0177	0.0189	0.0142	0.0142

**Table 4:** Potency matrix in iteration 2

	Expected cost	Probability of delay
Optimistic value	200,928	0.01
Pessimistic value	206,635	0.19

*Iteration 2:*

1. The potency matrix (Table 4) and the candidate solution are presented to the decision-maker: $f_1(a^{(3)}) = 205,194$; $f_2(a^{(3)}) = 0.04$.
2. The decision-maker is not satisfied with the proposal.
3. The decision-maker is not satisfied with the expected cost.
4. The set $\hat{\mathbf {A}}$ is identified:
  $$\begin{aligned} \hat{\mathbf {A}} := \left\{ a^{(4)}, a^{(5)}, a^{(6)}, a^{(7)}, a^{(8)}, a^{(9)} \right\} \end{aligned}$$
5. As the set $\hat{\mathbf {A}}$ is not empty, the procedure advances to the next step.
6. Trade-offs are calculated for $a \in \hat{\mathbf {A}}$ (Table 5). Alternative $a^{(9)}$ is identified as the new candidate solution. The procedure advances to step (10).
7. $\mathbf {A}^* := \hat{\mathbf {A}}$, $l:=3$, and the procedure advances to the next iteration.

**Table 5:** Trade-offs in iteration 2

Alternative	$a^{(4)}$	$a^{(5)}$	$a^{(6)}$	$a^{(7)}$	$a^{(8)}$	$a^{(9)}$
Trade-off	11.967	20.457	19.491	23.783	25.436	28.440

**Table 6:** Potency matrix in iteration 3

	Expected cost	Probability of delay
Optimistic value	200,928	0.10
Pessimistic value	204,476	0.19

*Iteration 3:*

1. The potency matrix (Table 6) and the candidate solution are presented to the decision-maker: $f_1(a^{(9)}) = 200{,}928$; $f_2(a^{(9)}) = 0.19$.
2. The decision-maker is not satisfied with the proposal.
3. The decision-maker is satisfied with the expected cost. The procedure advances to step (7).
4. The set $\hat{\mathbf {A}}$ is identified:
  $$\begin{aligned} \hat{\mathbf {A}} := \left\{ a^{(4)}, a^{(5)}, a^{(6)}, a^{(7)}, a^{(8)} \right\} \end{aligned}$$
5. As the set $\hat{\mathbf {A}}$ is not empty, the procedure advances to the next step.
6. Trade-offs are calculated for $a \in \hat{\mathbf {A}}$ (Table 7). Alternative $a^{(5)}$ is identified as the new candidate solution.
7. $\mathbf {A}^* := \hat{\mathbf {A}}$, $l:=4$, and the procedure advances to the next iteration.

**Table 7:** Trade-offs in iteration 3

Alternative	$a^{(4)}$	$a^{(5)}$	$a^{(6)}$	$a^{(7)}$	$a^{(8)}$
Trade-off	0.0254	0.0282	0.0189	0.0212	0.0142

*Iteration 4:*

1. The potency matrix (Table 8) and the candidate solution are presented to the decision-maker: $f_1(a^{(5)}) = 203,762$; $f_2(a^{(5)}) = 0.11$.
2. The decision-maker is satisfied with the proposal. The procedure advances to step (11).
3. End of the procedure.

**Table 8:** Potency matrix in iteration 4

	Expected cost	Probability of delay
Optimistic value	201,633	0.10
Pessimistic value	204,476	0.18

## Conclusion

In this paper the problem of determining the starting time of a non-critical activity is formulated as a bi-criteria dynamic decision making problem under risk. The main and original contribution of our work is a new interactive procedure that can be used for solving such problems. It uses trade-offs for identifying proposals for the decision maker. The procedure presented in the paper can also be used in other bi-criteria dynamic problems.

In the problem considered in this paper both criteria are minimized. However, the procedure presented in Sect. 3 can easily be adapted to the case where all or some of the criteria are maximized. In this case, it is necessary to modify the construction of the potency matrix. For the criterion whose higher values are preferred over the lower ones, the optimistic value is the maximum value, while the pessimistic value is the minimum value. It is also necessary to change the formula for calculating trade-offs. It is also obvious that in the case of the maximized criteria, it is necessary to change the formula, which allows to determine whether alternative *a* is dominated by $a'$. The rest of the procedure remains unchanged.

The problem considered in this paper is usually relatively small. In such cases it is possible to identify non-dominated alternatives using pair-wise comparisons. We start with the first alternative and compare it with all the others. Next, the second alternative is compared with all the others but the first one and so on. An alternative that is dominated by any of the others is eliminated and no longer compared. If the problem is larger (with more than 100 alternatives) and more than two criteria are considered, an algorithm using a quad-tree can be applied to speed up calculations (Habenicht 1992; Sun and Steuer 1996).

In our procedure the first candidate is an alternative that minimizes the expected cost. We assume that the decision-maker is primarily interested in optimising this criterion. However, it is possible to determine the first proposal in a different way. This solution can also be generated, for example, by using a rule based on the distance to the ideal solution, which is represented by the optimal values of all criteria. If both criteria are equally important for the decision maker, an alternative that is closest to ideal solution may be a good proposal.

We assume that the risk of delay is evaluated by an expert familiar with technical aspects of the activity. Such approach is usually used in practice, especially for engineering projects. The expert is able to assess the impact of various factors influencing the duration of the activity and provide quite a good estimation of the probability of delay. It is also possible to use more sophisticated methods to analyse the risk of delay. If the company is able to involve a group of experts, it is possible to use the Delphi method. The results obtained in this way are usually more reliable. However, it should be remembered that such analysis is time-consuming and expensive. If the company can afford this approach, it should definitely use it. Another approach that can be used for estimating the probability of delay is Monte Carlo simulation. This type of analysis usually provides very good results. However, its application is limited to a situation where estimates of the probability distributions of factors affecting the activity completion time are available. In practice, this condition is rarely met.

In our future research we will apply our approach to problems with more than two criteria. We will also consider using stochastic dominance rules, which make possible to analyse financial risk.
